# Slower Perception Followed by Faster Lexical Decision in Longer Words: A Diffusion Model Analysis

**DOI:** 10.3389/fpsyg.2015.01958

**Published:** 2016-01-05

**Authors:** Yulia Oganian, Eva Froehlich, Ulrike Schlickeiser, Markus J. Hofmann, Hauke R. Heekeren, Arthur M. Jacobs

**Affiliations:** ^1^Department of Education and Psychology, Freie Universitaet BerlinBerlin, Germany; ^2^Bernstein Center for Computational Neuroscience Berlin, CharitéBerlin, Germany; ^3^Dahlem Institute for Neuroimaging of Emotion, Freie Universitaet BerlinBerlin, Germany; ^4^Center for Cognitive Neuroscience, Freie Universitaet BerlinBerlin, Germany; ^5^Department of Psychology, Bergische Universitaet WuppertalGermany

**Keywords:** hierarchical diffusion model, lexical decision, length effect, bilingualism, grain size theory

## Abstract

Effects of stimulus length on reaction times (RTs) in the lexical decision task are the topic of extensive research. While slower RTs are consistently found for longer pseudo-words, a finding coined the word length effect (WLE), some studies found no effects for words, and yet others reported faster RTs for longer words. Moreover, the WLE depends on the orthographic transparency of a language, with larger effects in more transparent orthographies. Here we investigate processes underlying the WLE in lexical decision in German-English bilinguals using a diffusion model (DM) analysis, which we compared to a linear regression approach. In the DM analysis, RT-accuracy distributions are characterized using parameters that reflect latent sub-processes, in particular evidence accumulation and decision-independent perceptual encoding, instead of typical parameters such as mean RT and accuracy. The regression approach showed a decrease in RTs with length for pseudo-words, but no length effect for words. However, DM analysis revealed that the null effect for words resulted from opposing effects of length on perceptual encoding and rate of evidence accumulation. Perceptual encoding times increased with length for words and pseudo-words, whereas the rate of evidence accumulation increased with length for real words but decreased for pseudo-words. A comparison between DM parameters in German and English suggested that orthographic transparency affects perceptual encoding, whereas effects of length on evidence accumulation are likely to reflect contextual information and the increase in available perceptual evidence with length. These opposing effects may account for the inconsistent findings on WLEs.

## Introduction

The cognitive processes underlying visual word processing have been the target of intensive psycholinguistic research for many decades (Jacobs and Grainger, 1994; Norris, 2013). It is generally accepted that script is processed on two parallel routes of processing. On the lexical route letter representations are mapped directly to lexical word-form representations, whereas on the sublexical route orthographic signs are mapped to sublexical phonology by means of grapheme-to-phoneme associations (Coltheart et al., 2001; Perry et al., 2007). The increase in response times (RTs) with stimulus length, coined the word length effect (WLE), is thought to arise during sublexical processing (Barton et al., 2014). It is commonly interpreted as reflecting serial mapping in the sublexical route, requiring more time as an input contains more graphemes.

The two tasks most frequently used for the investigation of single word reading are naming and lexical decision (Jacobs and Grainger, 1994; Grainger and Jacobs, 1996). In naming, RTs are found to increase with stimulus length, seen as a consequence of serial encoding in the sublexical route, in particular for non-words, for which no lexical phonology exists (Perry et al., 2007). Although there have been different proposals as to the exact locus of the length effect within the sublexical route, in particular early visual encoding (Nazir et al., 1991; O'Regan and Jacobs, 1992; Jacobs et al., 2008), letter-to-grapheme mapping in the CDP+ model (Perry et al., 2007), or grapheme-to-phoneme mapping in the dual-route model (Coltheart et al., 2001), all models converge on the necessary activation of phonological representations in this task as the source for length effects (Hudson and Bergman, 1985; Ziegler et al., 2001b; Ferrand et al., 2011).

The findings on length effects in the lexical decision task, in which participants are presented with words and pseudo-words and are required to indicate the lexicality of a stimulus via button press, are less consistent. While an increase in RTs with length is found for pseudo-words (Ziegler et al., 2001a), most studies find no effects of length for real words. This was taken as evidence for the dominant contribution of the lexical reading route that was seen as not affected by length (Frederiksen and Kroll, 1976). Later evidence of inhibitory length effects for real words, however, challenged this notion (Balota et al., 2004). It was suggested that early stages of visual encoding might be affected by stimulus length due to reduced quality of visual input in the periphery (O'Regan and Jacobs, 1992).

Some studies even reported a decrease in RTs with length for words of up to six letters and increase in RT with length for longer words (O'Regan and Jacobs, 1992; New et al., 2006). This equivocal evidence suggests that the word length during lexical decision might be influenced by additional factors that were not considered so far. One possibility is that word length might take unique effects on different levels of processing during the lexical decision task. However, no systematic decomposition of the length effect in lexical decision has been conducted so far.

Another factor that affects the magnitude of the length effect on grapheme-to-phoneme mapping is orthographic transparency. Ziegler et al. (2001b) observed a larger effect of length on naming RTs in a transparent orthography with consistent letter-to-phoneme mapping (German) than in an inconsistent orthography (English). In line with this finding, the influential grain size theory (Ziegler and Goswami, 2005) states that in languages with high letter-sound consistency (transparent orthographies) letters are mapped to sound one-by-one, leading to strong effects of length on the speed of encoding. In languages with low letter-sound consistency, though, larger chunks of letters are mapped to sound simultaneously, leading to a reduction in length effects, as additional letters do not necessarily lead to additional steps in the encoding process. Support for this theory has been provided from native speakers of languages with varying degrees of orthographic consistency (Frost, 2006; Rau et al., 2015). However, it has not been investigated so far to what extent bilinguals are able to adjust their grapheme-to-phoneme encoding strategy to the demands of each of their orthographies.

The findings described above not only suggest that word length affects lexical decisions at several levels of processing, but also that these effects might vary with contextual factors, such as stimulus list composition or language. However, conventional analyses of lexical decision data, typically analysis-of-variance or multiple regression, are limited in their ability to isolate the (sub)-processes affected by a certain variable, such as length. This is not only because they focus on mean RTs and accuracies, which present the final outcome of the joint operation of these assumed sub-processes, but also because RTs and accuracies are mostly analyzed separately, and not as the joint outcome of the decision process (but see Grainger and Jacobs, 1996 for exceptions). A general alternative for the analysis of speeded 2-alternative decision tasks, such as lexical decisions, is the diffusion model framework, which estimates the unique contribution of a few separate sub-processes to a decision process described by the two-dimensional distribution of continuous RTs and binomial decisions (Busemeyer and Diederich, 2010). Here we apply this approach to the effects of word length on lexical decision behavior, with the aim of directly isolating the length effects on the sub-processes into which the diffusion model decomposes decision making.

**Figure 1:**
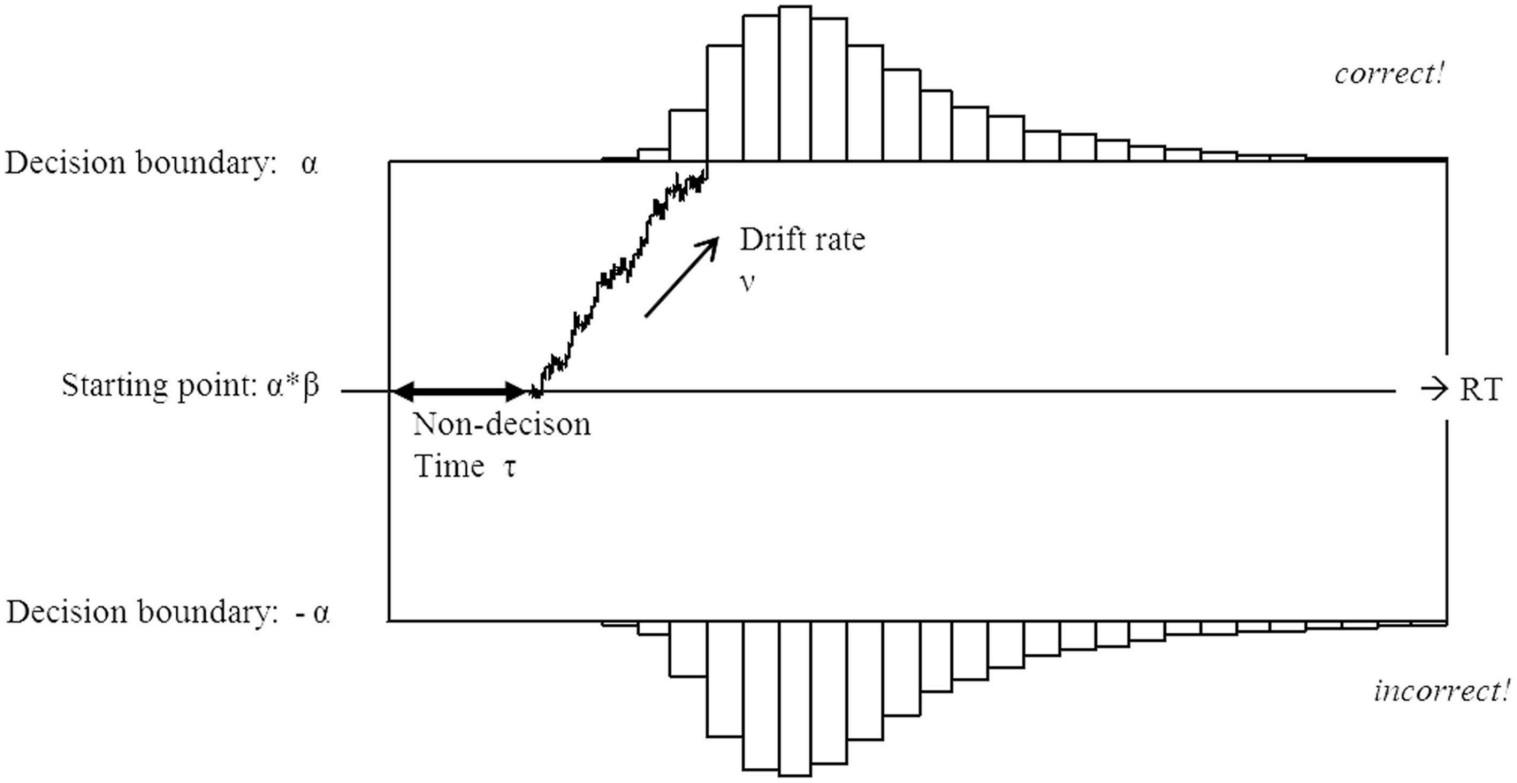
**Schematic illustration of the diffusion model**. The decision is made based on evidence accumulated with the drift rate ν. The average drift rate is positive on trials with the upper boundary being the correct response, and negative on trials with the lower boundary being the correct response. In this scheme the upper boundary represents the correct response. Non-decision processes, such as stimulus encoding and motor preparation are contained in the non-decision time τ. The decision is made once the amount of evidence exceeds one of the pre-defined decision boundaries α or 0. An unbiased decision process starts at the point $\frac{\alpha }{2}$, with a bias β of 0.5. A β larger than 0.5 indicates a bias toward the upper decision boundary, and a β smaller than 0.5 indicates a bias toward the lower decision boundary.

The diffusion model conceptualizes a decision between two alternatives as based on accumulation of evidence toward one of the decision alternatives (Ratcliff and McKoon, 2008; Voss et al., 2013). This accumulation is modeled using a decision variable, which drifts between two boundaries until it hits one of them, in which case the corresponding decision is made (absorbing boundaries). The drift process is characterized by four parameters (Figure 1): an initial bias toward one of the alternatives (β), the total time devoted to general, non-decision processes (τ, which include perceptual encoding and motor preparation), the rate of evidence accumulation (ν), and the distance between decisions boundaries (α). In this setup the upper boundary is located at α, and the lower boundary at 0. The final reaction time equals to the sum of the decision time and the non-decision time τ. The random variability of the decision process is represented as within-trial variability in the rate of evidence accumulation, which is not fixed in the model but is sampled from a normal distribution with mean ν and variance *s*. The latter is an intrinsic parameter of the model and changes in this parameters result in scaling of the other parameters. The joint distribution of total reaction time and the choice of a decision alternative in the diffusion model follows the Wiener distribution with parameters β, τ, ν, and α (see Vandekerckhove et al., 2011 for the exact mathematical form of the distribution). The multiple parameters of the diffusion model for a given data set can be obtained numerically by fitting the model to the data using maximum likelihood (Vandekerckhove and Tuerlinckx, 2008) or Bayesian estimation methods (Van Ravenzwaaij and Oberauer, 2009).

The diffusion model (DM) has several statistical advantages over the standard analysis of variance, such as being especially suitable to fit the typical left-skewed form of RT distributions (Van Breukelen, 1995), and the simultaneous mapping of continuous RTs and binomial response choices to one set of parameters, whereas in regression approaches, separate regression coefficients are estimated for RT and accuracy. However, a more major advantage is that the model parameters putatively reflect specific, cognitively meaningful, elements of the decision process. The drift rate (ν) conceptualizes the core of the decision process, namely the rate of evidence accumulation, which is larger when the amount of decision-relevant information in the stimulus is larger. The non-decision time (τ) is associated with stimulus encoding and response-unspecific motor preparation. Importantly, for decisions with little variance in motor preparation (i.e., when only a simple button press is required), increases in non-decision time are usually attributed to more effortful stimulus encoding (Ratcliff and Smith, 2010). The decision boundary (α) is associated with the amount of evidence necessary for a decision, such that higher boundary values stand for more conservative decision strategies, and lower values for fast but less accurate decisions (explaining the commonly observed speed-accuracy trade-off (Bogacz et al., 2006; Wenzlaff et al., 2011). Finally, the bias parameter is associated with the a-priori tendency in favor of one of the two alternatives. These relative advantages have led to successful applications of the diffusion model to the lexical decision task (Ratcliff et al., 2004a; Wagenmakers et al., 2004, 2008). Most notably, the diffusion model was used to map well-known effects on RTs to selective changes in certain parameters, while showing that all other parameters remained unchanged. For example, changes in stimulus list composition were shown to induce shifts in decision boundary (Wagenmakers et al., 2008), and differences in word frequency to change drift rates (Ratcliff et al., 2004a).

The standard approach to fitting the DM is to estimate the parameters separately for each participant in each condition, which, however, requires a large number of data points (>200) to achieve stable parameter estimations (Ratcliff and Tuerlinckx, 2002). This is particularly problematic in fields such as psycholinguistics, where such large numbers of stimuli per experimental condition are not always possible to achieve. In the past, this problem was most often solved by pooling of data over a group of participants (“meta-subject,” Busemeyer and Diederich, 2010). However, this approach ignores inter-subject variability and thus does not allow statistical inference beyond a specific sample. Moreover, it provides only limited insight into factors determining the decision process, as it does not allow for analysis of inter-individual differences. Recently, a novel analytic approach was suggested, the hierarchical diffusion model (HDM, Vandekerckhove et al., 2011), which allows estimating DM parameters of single participants even in sparse data sets. In HDM, the implicit assumption that all participants of an experiment stem from one population is made explicit by assuming that for each parameter, values of all participants stem from an underlying normal distribution:

$$\begin{array}{l} \nu_{i}\sim \;N\left(\mu_{\nu },\;\sigma_{\nu }\right),\\ \tau_{i}\sim \;N\left(\mu_{\tau },\;\sigma_{\tau }\right),\\ \alpha_{i}\sim \;N\left(\mu_{\alpha },\;\sigma_{\alpha }\right),\\ \beta_{i}\sim \;N\left(\mu_{\beta },\;\sigma_{\beta }\right),\\ i=1,\ldots ,n\;participants. \end{array}$$

These constraints bind the parameter values of a single participant by the distribution defined by the parameters of all other participants, enabling simultaneous estimation of the parameters of all participants. While the simple DM (i.e., non-hierarchical DM for a single participant) is typically estimated using maximum-likelihood estimates, this is not possible due to the increased complexity of the HDM. However, HDM can be fit in a Bayesian approach using Markov-Chain Monte-Carlo (MCMC) approximations of the underlying distributions (for a detailed description of this approach see Vandekerckhove et al., 2011).

In the present study, we used HDM to investigate the cognitive locus of length effects in lexical decision, and to compare HDM parameters between first and second languages in German-English bilinguals. First, we were interested in identifying the differences in diffusion model parameters underlying slower RTs for foreign language words, as routinely found in bilingual lexical decision. We reasoned that if they were due to participants' more cautious decision making behavior in a foreign language, they would be reflected in an increase in the distance between decision boundaries only. If on the other hand, they were due to less stable and harder to access L2 lexical representations (Hsu et al., 2014), the effect would be reflected in a decrease in rate of evidence accumulation for the L2 as compared to L1, and possibly also an increase in non-decision times. Second, we expected the prolonging effect of length on RTs to pseudo-words to be selectively reflected in increases in non-decision times, which would be in line with the sublexical locus of the length effect, as sublexical representations are generally assumed to not contribute evidence to lexical decisions. If differences in the length effect between languages are due to difference in encoding strategies following orthographic transparency, this effect will differ between German and English language blocks. Finally, we were interested to see how the length of words would affect the non-decision time and drift rate parameters. The isolation of these effects would advance the localization of length effects on words to sublexical and lexical stages of models of visual word recognition.

## Methods

### Participants

This study was conducted with 28 native speakers of German with high proficiency in their second language English. All were students at the Freie Universitaet Berlin and had studied English as their first foreign language in high school. Participants were right-handed, had normal or corrected-to-normal vision, and reported no reading disability or other learning disorders. All participants completed an online language history questionnaire (adapted from Li et al., 2006) prior to participation, with self-reports of L2 proficiency on a 1–7 Likert scale, separately for reading, writing, speaking and listening abilities. Self-reports of L2-proficiency are summarized in Table 1. Participants were recruited through advertisements on campus and in mailing lists for experiment participation. All participants completed an informed consent form prior to beginning the experiment. They were reimbursed either monetary or with course credit. The experiment was approved by the ethics board of the Psychology Department of the Freie Universitaet Berlin.

**Table 1:** **Summary of participants' foreign language proficiency and reading ability in German (L1) and English (L2)**.

***n* = 28 (17 female)**		**Age**	**Mean**	***SD***
			**24.8**	**4.5**
Self-report of L2 proficiency		Reading	5.5	0.8
		Writing	4.7	1.0
		Speaking	4.8	1.3
		Listening	5.2	1.2
		Accent	3.1	1.4
		Years spoken	16.8	6.8
Reading rate	German	Words	117.4	23.7
		Pseudo-words	73.5	21.2
	English	Words	81.3	12.5
		Pseudo-words	57.0	8.5
Lextale	German		90.4	4.9
	English		72.7	11.2

### Assessment of language skills

In addition to the pre-experimental screening questionnaire, participants' general proficiency was also assessed after the experiment using the LEXTALE tests of German and English proficiency (Lemhöfer and Broersma, 2011). The tests consist of short lexical decision tasks, which include words of varying frequency and pseudo-words. The final score is the average percentage of correct responses to words and pseudo-words. Reading abilities were assessed using the reading and phonological decoding subtests of the TOWRE (Torgesen et al., 1999) for English, and the word and pseudo-word reading subtests of the SLRT-II test (Moll and Landerl, 2010) for German. Both tests assess reading rate (words/min) in single-item reading of words and pseudo-words (PW). Participants' language profile is summarized in Table 1.

### Stimuli and design

The stimulus set contained German and English words and pseudo-words of length 3–6, covering the range for which contradictory findings were reported previously (New et al., 2006; Ferrand et al., 2010). Words were chosen based on frequency counts in the SUBTLEX corpus (New et al., 2007; Brysbaert et al., 2011). For each language and length there were 31 words and 31 pseudo-words, resulting in 124 words and 124 pseudo-words per language. Pseudo-words were created from the words by changing 1–2 letters in random positions while retaining orthographic legality in the respective language. Stimuli were matched at the group level across languages (German/English), stimulus types (word/pseudo-word), and length (3–6) on word frequency (log10 word frequency normalized per million words, words only), orthographic neighborhood size (average Levenshtein distance to 20 nearest orthographic neighbors, *OLD20*, Yarkoni et al., 2008), and mean bigram frequency (log10 non-positional bigram frequencies, normalized per million bigrams). Orthographic neighborhoods differ between German and English due to a different distribution of word lengths in the two languages. Thus, OLD20 was normalized within language and stimulus length prior to matching (for a similar procedure see Oganian et al., 2015). Moreover, stimuli had 1–2 syllables and were matched in syllable numbers across languages and stimulus types.

A summary of stimulus properties can be found in Table 2, and the complete list of stimuli is presented in the Supplementary Material.

**Table 2:** **Ranges of word frequency, orthographic neighborhood, and bigram frequency for German and English words and pseudo-words**.

		**log10 word frequency**	**OLD20**	**log10 bigram frequency**	**Syllable number (mean, SD)**
German	Words	[0.95, 2.5]	[1, 2.8]	[3.2, 4.3]	1.18 (0.4)
	Pseudo-words	–	[1, 2.8]	[3.3, 4.3]	1.18 (0.4)
English	Words	[1.08, 2.8]	[1.1, 2.7]	[3.2, 4.3]	1.35 (0.5)
	Pseudo-words	–	[1, 2.7]	[3.3, 4.2]	1.34 (0.5)

### Procedure

Participants performed a block of German lexical decision and a block of English lexical decision in counterbalanced order. In each trial of the lexical decision task, participants were required to indicate whether a visually presented letter string was an existing word in the language of the block. Each stimulus was presented for upmost 2 s or until a response was given. Between trials a fixation cross was presented for 800 ms. Each block consisted of 248 trials, resulting in about 8 min per block. Each block was preceded by a short text reading exercise (English: 1159 words; German: 1095 words) to ensure immersion in the respective language (see Elston-Güttler et al., 2005 for a similar procedure). After each block participants conducted the reading and proficiency tests for the respective language. The overall duration of the experiment was 45 min.

### Data analysis

#### Outlier exclusion

RT-based outliers were defined as raw reaction times outside 2.5 SD of participants' mean in each language X stimulus experimental cell. Moreover, we excluded items for which over 40% of participants gave incorrect responses, to ensure that word stimuli (most importantly foreign language words) were known to participants, resulting in exclusion of 22 items (4.5% of the stimulus set).

#### Analyses of mean reaction times and accuracies

To provide a comparison between analyses of mean RTs and accuracies and HDM statistics we conducted an analysis of mean % error and RTs using mixed-effects modeling (LME, Baayen et al., 2008) with crossed random factors for subjects and items. Mean reaction times were analyzed using linear mixed-effects regression, which included main effects and interactions for language, stimulus type, and length as fixed factors. Mean % error was analyzed using logistic mixed-effects regression. Due to the low numbers of errors in lexical decision tasks, error analysis included only language and stimulus type as fixed factors. The random factor structure included random intercepts for items, as well as random intercept and random slopes for the highest order interaction of language, stimulus type, and length for subjects, as recommended by Barr and colleagues (Barr, 2013; Barr et al., 2013).

#### Hierarchical diffusion modeling

We fitted the RT and accuracy data with an HDM (Vandekerckhove et al., 2011), as outlined in the introduction. We made two further assumptions to simplify the model. First, as each block contained equal numbers of words and PWs and previous studies did not report systematic biases toward either response in similar setups (Ratcliff et al., 2004a,b), we assumed a symmetrical drift process and hence set the bias parameter (β) to 0.5. Second, while some versions of the diffusion model include random variation in mean drift rate and non-decision time between trials (Voss et al., 2013), we set these inter-trial variances in drift rates and non-decision times to 0 (for a similar approach see Krypotos et al., 2014)^1^.

In the hierarchical approach the diffusion model parameters are simultaneously estimated for all participants and all conditions. Thus, it is necessary to define within the model the relationship between experimental variables and model parameters, which we did based on our theoretical assumptions. The decision boundary (α) was allowed to vary across participants and to differ between the English and the German block in each participant, such that two decision boundary values were estimated in each participant. This allowed us to investigate whether participants were more conservative in lexical decisions in a foreign language. Second, as the main aim of our study was to investigate the effects of length on non-decision time (τ) and drift rate (ν), we parameterized τ and ν as linear functions of stimulus length. This was done by constraining mean values for both parameters in each language X stimulus type condition to vary linearly with length by means of linear link functions with:

$$\begin{array}{l} \tau_{pgw}(\text{length})={\text{t}}_{pgw}^{0}+\text{length}\cdot {\text{t}}_{pgw}^{1},\\ \nu_{pgw}(\text{length})={\text{v}}_{pgw}^{0}+\text{length}\cdot {\text{v}}_{pgw}^{1}, \end{array}$$

*p* = 1, …, n (participants); *w* = 1, 2 (word/pseudo-word); *g* = 1, 2 (German/English).

In these link functions the intercepts (t^0^ and v^0^) are the average values of non-decision time and drift rate across all possible length values, whereas the slopes (t^1^ and v^1^) reflect the effect of word length (as measured by the number of letters). A slope of 0 would mean no effect of length, a positive value an increase in RT with length, and a negative value a decrease of RT with length. Single participants' parameters $\left(t_{pgw}^{0},t_{pgw}^{1},\;v_{pgw}^{0},\;v_{pgw}^{1}\right)$ were fitted under the assumption that they stem from normal population distributions, whose means were allowed to vary as function of stimulus type and language (see Supplementary Table S1 for the JAGS code definition of the model). An additional assumption was that population-level distributions of each parameter would have equal variances across language X stimulus type conditions, rendering the estimation of variance more stable. In summary, for each participant we estimated the posterior distributions of 18 parameters: eight drift rate parameters, eight non-decision time parameters (two per stimulus type and language), and two alpha values (one per language). We fitted the diffusion model with within-trial variability of the drift rate *s* = 1, as it is implemented in R (Wabersich and Vandekerckhove, 2014b) and JAGS (Wabersich and Vandekerckhove, 2014a).

#### Bayesian model estimation

We estimated the parameters of the HDM using a Bayesian approach. Within this approach, each parameter of the model is assigned with a prior probability distribution, describing the possible range of parameter values. The posterior distribution of each parameter given the data and all other parameters can be approximated based on Bayes' theorem. The mean of the posterior distribution is then used as an estimate of the parameters' values given the data. The Bayesian framework naturally integrates a hierarchical model structure, estimating each parameter based on the constraints provided by higher-order hierarchies. The posterior distributions can be easily estimated using the numerical algorithm known as Markov-chain Monte-Carlo sampling (MCMC, for an introduction to Bayesian and MCMC see Kruschke, 2010). MCMC is a method that sequentially samples values for each parameter, resulting in a “chain” of parameter values, which can be used to construct a posterior distribution for each parameter. Convergence and stationarity are typically assessed by means of several quality checks. First, it is important to run several MCMC chains and to visually ensure that all chains converge to the same posterior distributions. Second, the Gelman-Rubin statistic (R-hat) provides a measure of chain stationarity. This statistic should be <1.1 in a stationary chain (Gelman and Hill, 2007).

Here, MCMC sampling was conducted in the freely available software package JAGS (Wabersich and Vandekerckhove, 2014a), via the RJAGS library for R (Plummer, 2014). Convergence was assessed using functions from the coda package for R (Plummer et al., 2006). We ran 2 MCMC chains with starting values based on a simplified version of the diffusion model for which closed-form expressions for drift rate and non-decision time exist (EZ-model, Wagenmakers et al., 2007) and uninformative uniform priors for group-level means and variances of all parameters. Each chain contained 10,000 samples after a burn-in period of 1000 samples. For the resulting chains we assessed convergence and stationarity using the Gelman-Rubin statistic and by visually inspecting the chains.

Finally, we used posterior estimates of the group level parameters to simulate data for each of our conditions. Simulated data were used to validate the model through a comparison to the empirical data.

#### Statistical inference

To examine the effects of stimulus type and language on drift rate, non-decision time, and decision boundary, we submitted the means of single participants' posterior distributions of each of these parameters to within-subject analyses of variance (ANOVAs). To investigate whether drift rate and non-decision time were significantly modified by stimulus length we used simple *t*-tests against 0 on the slopes of non-decision time and drift rate (t^1^, v^1^). This corresponds to the null hypothesis of no effect of length, in which case the slopes would be equal to 0. For the ANOVA analyses we quantified effect sizes as generalized eta squared (${ɳ}_{G}^{2}$, Bakeman, 2005).

## Results

Based on the outlier exclusion criterion 6% of trials were excluded, but not more than 7% per participant.

### Regression analysis of mean RTs and accuracies

The linear mixed-effects analysis of reaction times (Table 3) showed that reaction times increased with length, *b* = 21.73, *SD* = 4.3, *t* = 5.1, χ_(1)_ = 25.6, *p* < 0.001. RTs were also higher for pseudo-words than for words, *b* = 74.9, *SD* = 6.8, *t* = 11.0, χ_(1)_ = 121.9, *p* < 0.001, and in the English block than in the German block, *b* = 74.67, *SD* = 6.8, *t* = 10.9, χ_(1)_ = 121.0, *p* < 0.001. The interaction of stimulus type and length, *b* = −20.4, *SD* = 6.1, *t* = −3.4, χ_(1)_ = 11.2, *p* < 0.001, reflected that mean RTs were not affected by length for words (*p* = 0.76), but that they increased with length for pseudo-words, *b* = 21.67, *SD* = 4.2, *t* = 5.12, χ_(1)_ = 26.3, *p* < 0.001 (see **Figure 3A**). The effects of length and stimulus type did not interact with language, i.e., the pattern of effects was similar for both languages.

**Table 3:** **Mean reaction times and error rates**.

		**Reaction times [ms] (SD)**				**%Error**	
		**Correct**		**Error**			
German	Words	614	(33)	654	(40)	3.1	(3.6)
	Pseudo-words	690	(37)	624	(50)	3.8	(3.3)
English	Words	675	(38)	850	(59)	5.9	(4.8)
	Pseudo-words	762	(42)	852	(55)	6.9	(4.5)

The logistic mixed-effects analysis of % errors (Table 3) showed that participants made more errors in English than in German, *b* = 0.99, *SD* = 0.35, *z* = 2.8, *p* = 0.004. All other effects were not significant, although the number of errors was marginally larger for pseudo-words than for words, *b* = 0.56, *SD* = 0.31, *z* = 1.8, *p* = 0.06.

In summary, analysis of mean RTs and accuracies suggested that participants' were slower and more prone to errors in their L2 (English) than in their L1 (German). Moreover, they were slower for pseudo-words than for words. Importantly, the RTs analysis showed slower RTs with length for pseudo-words, whereas there were no effects of length for words.

### Hierarchical diffusion modeling

#### Assessment of convergence

**Figure 2:**
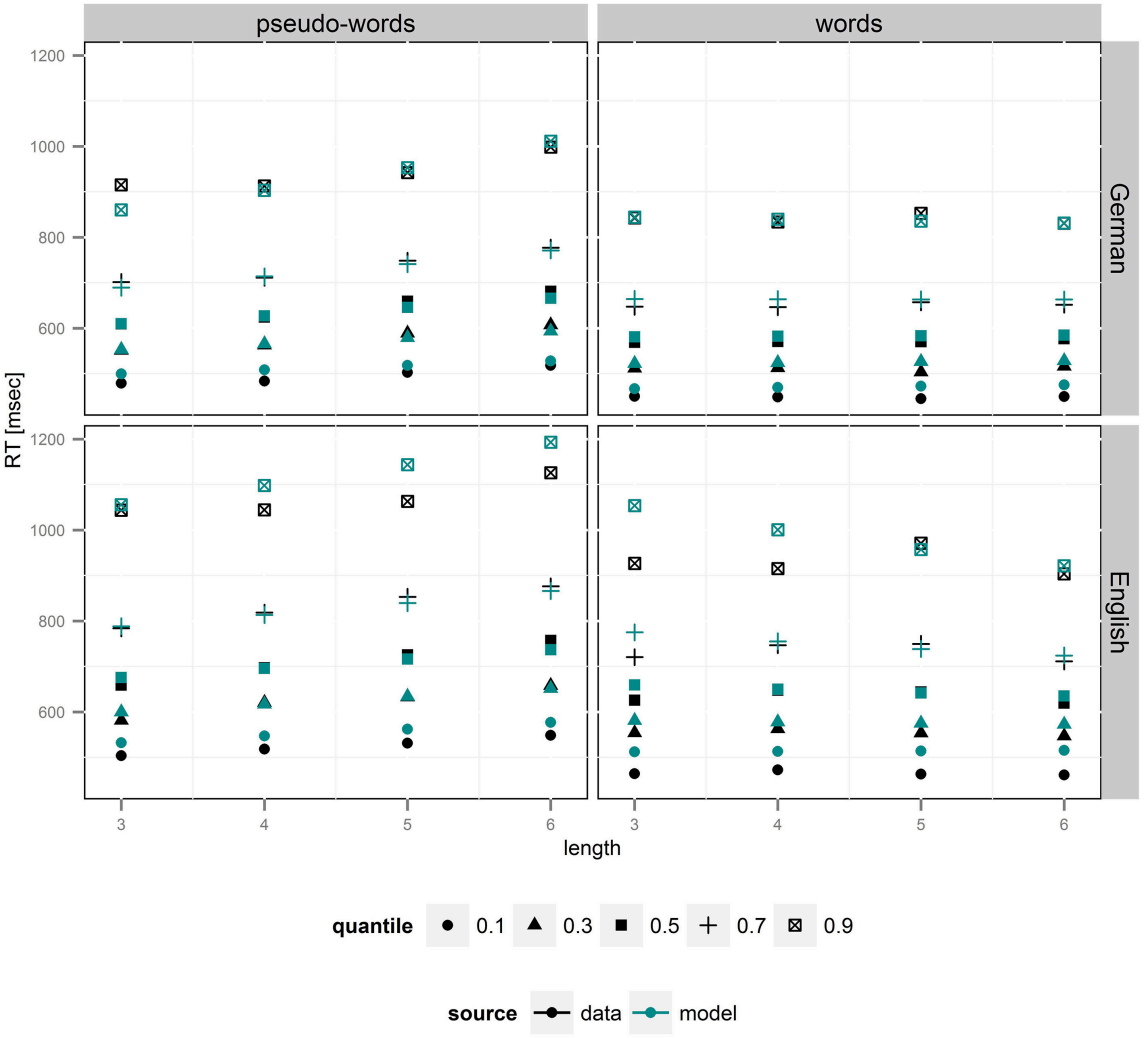
**Plot of group RT quantiles (***q*** = 0.1, 0.3, 0.5, 0.7, 0.9) for correct responses based on empirical data (black) and group-level diffusion model parameters (blue)**. Increase in non-decision time is reflected in an upward shift in shortest reaction times, which is strongest in pseudo-words. Lower drift rates, in contrast, lead to acceleration of slowest RTs, i.e., the right tail of the RTs distributions, an effect strongly present for German pseudo-words of different lengths.

As can be seen in Supplementary Figure S1, the R-hat statistic was below 1.02 for all variables, indicating successful convergence of the MCMC chains to stationary posterior distributions for all model parameters. Simulations of RT distributions showed that the model fitted our data very well, as the correlation between empirical and model RT quantiles was *r* = 0.98 (Figure 2). Importantly there were no systematic deviations between empirical and model RT distributions.

#### Analysis of posterior estimates of DM parameters

**Figure 3:**
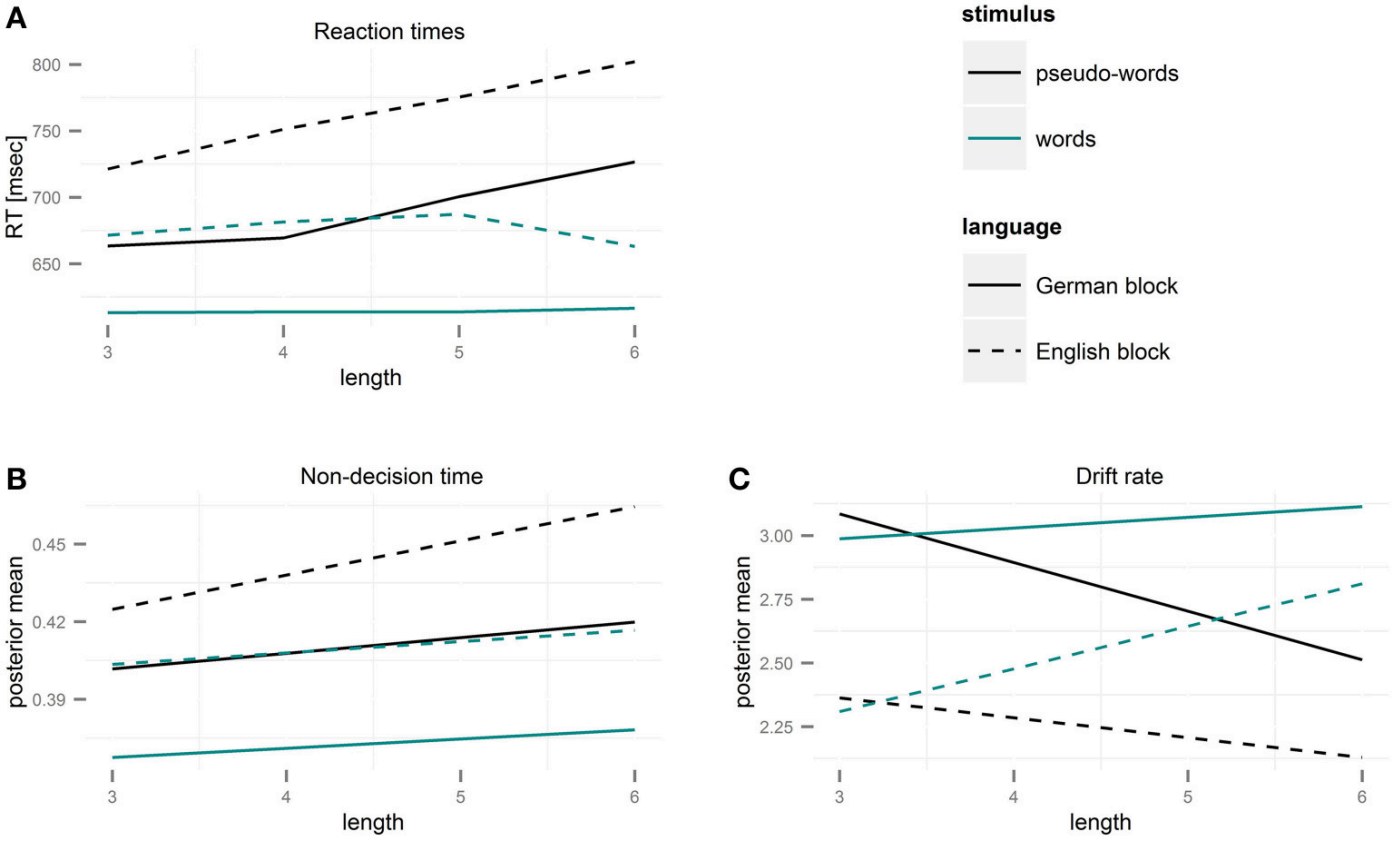
**Length-induced changes in drift rate and non-decision time as function of stimulus type and language**. **(A)** Reaction times for correct responses to words and pseudo-words in German and English. RTs increase for PWs but not for words with length. **(B)** Estimates of non-decision times for words and pseudo-words in German and English based on parameters of the diffusion model. Non-decision time increased with length in all four conditions. **(C)** Estimates of drift rates for words and pseudo-words in German and English based on parameters of the diffusion model. Evidence accumulation slowed down with length for pseudo-words, but was faster for long than for short words.

The intercept and slope parameters for drift rate (v0, v1) and non-decision time (t0, t1) of each participant were subjected to repeated-measures ANOVAs with factors language and stimulus type. The boundary parameter (α) was subjected to a paired *t*-test to assess differences between German and English blocks. Mean posterior parameter values are listed in Table 4, and the effects of length on non-decision time, and drift rate are presented in Figure 3, where changes in RTs with length are also plotted for comparison.

**Table 4:** **Posterior estimates for non-decision time, drift rate, and decision boundary**.

	**German**		**English**	
	**Pseudo-words**	**Words**	**Pseudo-words**	**Words**
Decision boundary (α)	1.55 [1.43; 1.66]		1.51 [1.42; 1.59]	
**NON-DECISION TIME**				
Intercept (t^0^)	0.41 [0.38; 0.44]	0.37 [0.35; 0.39]	0.44 [0.41; 0.47]	0.41 [0.39; 0.17]
Slope (t^1^)	0.006 [0.002; 0.01]	4·10^−3^ [4·10^−5^; 7·10^−3^]	0.013 [0.009; 0.02]	0.004 [8·10^−4^; 8·10^−3^]
**DRIFT RATE**				
Intercept (v^0^)	2.80 [2.54; 3.05]	3.05 [2.84; 3.25]	2.24 [1.95; 2.53]	2.56 [2.2; 2.92]
Slope (v^1^)	−0.19 [−0.2; −0.17]	0.04 [0.02; 0.06]	−0.08 [−0.09; −0.06]	0.17 [0.15; 0.18]

*Table displays means and 95% confidence intervals*.

##### Decision boundary

The decision boundary did not differ between German and English blocks (*p* = 0.4).

##### Non-decision time

The intercept of the non-decision time, t^0^, was larger in the English block than in the German block, *F*_(1, 27)_ = 14.44, *p* < 0.001, ${ɳ}_{\text{G}}^{2}=0.09$, and for pseudo-words than for words, *F*_(1, 27)_ = 28.18, *p* < 0.001, ${ɳ}_{\text{G}}^{2}=0.09$, indicating an overall increase in non-decision time in a foreign language and for novel letter strings (Figure 3B). The interaction effect of language and stimulus type was not significant. The slope of the change in non-decision time as function of word length, t^1^, was significantly larger in the English than in the German block, *F*_(1, 27)_ = 5.29, *p* = 0.03, ${ɳ}_{\text{G}}^{2}=0.04$, and for pseudo-words than for words, *F*_(1, 27)_ = 9.91, *p* = 0.004, ${ɳ}_{\text{G}}^{2}=0.08$, while the interaction effect of language and stimulus type was not significant. As we were interested in characterizing the magnitude of the length effect for each language and stimulus type separately, we conducted planned *t*-tests to test whether the slope was significantly different from 0 in each of the four conditions. Indeed, this was true for all cases [German words: *t*_(27)_ = 2.08, *p* = 0.05; German PWs: *t*_(27)_ = 2.98, *p* = 0.005; English words: *t*_(27)_ = 2.52, *p* = 0.02; English PWs: *t*_(27)_ = 7.56, *p* < 0.001]. In summary, we found an increase in non-decision time with length for words and pseudo-words in both languages, with stronger effects in the foreign language and for pseudo-words.

##### Drift rate

The intercept of the drift rate, v^0^, was larger in the German block than in the English block, *F*_(1, 27)_ = 25.68, *p* < 0.001, ${ɳ}_{\text{G}}^{2}=0.12$, and for words than for pseudo-words, *F*_(1, 27)_ = 12.62, *p* = 0.001, ${ɳ}_{\text{G}}^{2}=0.04$. The interaction of language and stimulus type was not significant. The effect of length on the drift rate, reflected in the slope v^1^, was larger in the English block than in the German block, *F*_(1, 27)_ = 224.62, *p* < 0.001, ${ɳ}_{\text{G}}^{2}=0.70$, and for words than for pseudo-words, *F*_(1, 27)_ = 902.42, *p* < 0.001, ${ɳ}_{\text{G}}^{2}=0.9$. Importantly, this was due to negative values of t^1^ for German and English pseudo-words (*p*'s < 0.001 for *t*-test against 0), and positive values of ν^1^ for words (*p*'s < 0.001 for *t*-test against 0). Thus, drift rates decreased with length for pseudo-words but increased for words in both languages (Figure 3C).

## Discussion

The present study examined the length effect in lexical decision in German-English bilinguals using a diffusion model (DM) analysis. Participants made English and German lexical decisions on 3–6 letter words and pseudo-words. The DM allowed us to distinguish between decision-unrelated perceptual encoding and evidence accumulation toward one of the decision alternatives. Hierarchical Bayesian estimation of the diffusion model parameters allowed for a stable fit at the single subject level. The results of this approach were contrasted with a linear mixed-effects regression analysis of mean RTs.

Across word lengths, lexical decisions were faster and more accurate in participants' native language German (L1) than in their second language English (L2). The HDM revealed that this difference was selectively associated with increased non-decision times and slowed evidence accumulation, but not with modification of decision boundaries. This finding implies that longer lexical decision times in a second language are likely to result from less efficient stimulus encoding and lexical activation, rather than more conservative decision making (which would be manifested in higher decision boundaries, Ratcliff et al., 2004b; Busemeyer and Diederich, 2010). Differences between pseudo-words and words were similar across languages, with converging evidence from mean-based regression and diffusion model analyses. Namely, RTs were longer for pseudo-words than for words, in agreement with higher non-decision times and smaller drift rates for pseudo-words than for words.

Mean RTs and accuracies showed no length effect for word stimuli, whereas response times to pseudo-words increased with length, in concordance with previous studies (Ziegler et al., 2001b; Martens and de Jong, 2006; Yap et al., 2012; see New et al., 2006, Table 1 for a summary of previous studies of the length effect in lexical decision). However, the DM, which helps decomposing the decision making process into constituent sub-processes, provided a more differentiated picture. Namely, while the effects of length on non-decision time and drift rate for pseudo-words were inhibitory, as expected, the pattern for words was different. In contrast to the overall null effect of length on word RTs, we found that while perceptual encoding became slower with length, evidence accumulation accelerated for words of both languages, as evident by increased non-decision times as well as faster drift rates with length. This novel pattern offers an explanation for the contradicting patterns previously reported, namely that length effects on lexical decision RTs reflect the interaction of several opposite sub-processes, such that the overall effect depends on the relative strength of each of the single effects.

Our findings of length effects on non-decision times in German and English provide novel insights with respect to two aspects of the reading process. First, dual-route models of reading aloud (e.g., DRC: Coltheart et al., 2001; CDP+: Perry et al., 2007) usually argue that mapping of orthographic input on the lexical route is not affected by length. The length effects on non-decision times for real words in our data suggest differently, namely that visual and/or sublexical encoding of letter strings is affected by length even in the lexical route. This could be due to reduced peripheral visual quality of encoding, which would have strongest effects on long words (Nazir et al., 1991; Jacobs et al., 2008). Alternatively it could be that letter to grapheme encoding as postulated for the sublexical route in the CDP+ model also happens in the lexical route. Second, the grain size theory (Ziegler and Goswami, 2005) predicts that for native speakers non-decision times will be affected by length in German more than in English, as encoding of letter strings and mapping to phonological units will be slowed by length in the transparent German orthography more than in the deep English orthography, due to a larger number of graphemes in a letter string. However, they make no clear predictions regarding bilinguals' encoding strategies. Our diffusion model analysis shows that the effect of length on non-decision times was equal in German and English for words, but larger in English than in German for pseudo-words. We suggest that our participants might in fact be using smaller than optimal grain sizes to encode English words, which is more effortful due to the ambiguous mapping between small letter clusters and phonology in English, requiring more time than in German. This result implicates that bilinguals do not gain full control over the use of different grain sizes when switching between languages, despite their high proficiency in reading the second language.

One theoretical explanation for the acceleration of evidence accumulation with length for words is along the lines of the recently reported decreases in RTs with length for short words (i.e., 3–6) and increase for longer (>8 letters) words (New et al., 2006; Ferrand et al., 2010, 2011). New et al. (2006) analyzed a large set of English lexical decision data and found a decrease in RT with length for 3–6 letter long words. They argued that the facilitatory effect of length for short words could be partially due to eye movement patterns during reading, as well as the fact that word length 3 is less frequent, and thus less expected, in English than word length 6. Ferrand et al. (2010) found a slight decrease in RTs with length for monomorphemic French words when they were presented in the context of polymorphemic words, but no length effect when words were presented in a monosyllabic stimulus set. Based on their findings, New et al. and Ferrand et al. argued that processing of words of an expected length is faster than for words of untypical lengths, whereby expectations could stem from the specific stimulus list composition as well as from general statistical patterns in the language under study. The comparison between length effects in English and German in our data supports this proposal. Namely, we find a larger facilitation of evidence accumulation by length for English words than for German words. As 5–7 is the most frequent word length in English, whereas German words are longer on average (9–11 letters in the SUBTLEX), the peak of the facilitatory effect in German might be reached at a greater length than in our stimulus set. Importantly though, the facilitative effects of word length in our German stimuli suggest that this phenomenon is not limited to the two languages tested in these studies. An important question for further (simulation-) studies is whether faster accumulation of lexical activation with length for words can be accounted for by models of visual word recognition.

What information is accumulated toward a response in lexical decision? The answer is well established for “yes”-responses to words, which are typically assumed to reflect the similarity of the input to lexical representations, i.e., the amount of activation in the lexical network (e.g., in the MROM, Grainger and Jacobs, 1996, or in the Bayesian Reader, Norris, 2006). The case is, however, less clear for “no”-responses to pseudo-words, for which the absence of positive information is decisive. A prominent model is the deadline model, which assumes that “no”-decisions are made when positive evidence was not sufficient within a certain time period (Grainger and Jacobs, 1996). While the deadline model is plausible cognitively, it has been criticized for its failure to account for a reversal of the RT pattern for yes/no-responses under certain experimental conditions (Wagenmakers et al., 2008). A recent suggestion to solve these issues is that evidence accumulated toward a “no”-response equals to some constant (e.g., the expected amount of evidence for a “yes”-response) minus the amount of evidence for a “yes”-response (Leaky accumulator model, Dufau et al., 2012)^2^. Importantly, one of the predictions of this coupling of drift rates for words and pseudo-words within one stimulus block is that if the drift rate for words increases in one condition, it should decrease for pseudo-words of the same condition. Interestingly this is exactly the pattern that we see for different word lengths: while the drift rate for words increased with length, the drift rate for pseudo-words decreased with length. However, in our experiment drift rates for words and pseudo-words do not sum up to a constant value across different lengths, but rather show an overall decrease with length. Whether this can be accommodated in the framework proposed by Dufau et al. possibly by means of an evidence-based adjustment of expected lexical evidence should be the topic of future (simulation) studies.

A major difference between our results and previous reports (New et al., 2006; Ferrand et al., 2011) of facilitatory effects of length on lexical decision is that the effect in our data is only apparent on the level of DM parameters and not in raw RTs. A definitive explanation of these different patterns would require further comparisons between regression approaches and DM results. However, as our data suggest that the overall effect on mean RTs results from interplay of facilitation at the level of drift rates and inhibition in non-decision time, we can speculate that these two effects may be differently affected by contextual or language-specific factors. The overall pattern of RTs could then vary from inhibitory effects to facilitatory, as has been reported in the past (inhibitory effects: O'Regan and Jacobs, 1992; null effects of length in lexical decision: Frederiksen and Kroll, 1976; facilitatory effects: Ferrand et al., 2011).

The diffusion model approach has major advantages over conventional measurement models of mean RTs. By fitting the complete RT distributions for correct and erroneous responses, the model estimates several latent subcomponents of the decision process. It provides thus novel predictions that can be used to advance the development of computational process models of visual word recognition (Jacobs and Grainger, 1992; Grainger and Jacobs, 1996; Perry et al., 2010). In particular, the results of the present study demonstrate that conventional analysis methods are too crude to provide a sufficient resolution for understanding of length effects, which require a mathematical decomposition of the underlying processes. This can inspire future research using full process modeling of the decision process (e.g., Grainger and Jacobs, 1996; Perry et al., 2007; Hofmann and Jacobs, 2014). Moreover, the hierarchical approach to diffusion modeling adopted here (Vandekerckhove et al., 2011; Krypotos et al., 2014) allows fitting the diffusion model at the single subject level, despite the sparseness of the data set. This approach enables the comparison between parameters of the diffusion model in different conditions and an analysis of individual differences in small and medium sized data sets—an endeavor that until now was limited to large scale projects, such as the English lexicon project (Yap et al., 2012). We provide the example code for defining and fitting our model in the supplementary online material, in the hope to provide valuable support for researchers with interest in diffusion modeling.

## Conclusion

The contradictory findings on the length effect in lexical decision have attracted a great deal of speculation. The magnitude and direction of the length effect have been associated with factors such as orthographic depth, stimulus type, and contextual information in terms of stimulus list composition as well as statistical properties of the languages under study. Here we use a novel approach, hierarchical diffusion modeling, to tackle the effects of stimulus length on different subcomponents of the lexical decision process. Our findings shed light on the dual nature of length effects, disclosing an inhibitory effect on perceptual encoding but a facilitatory effect on lexical-activation-based decision. Moreover, our data provide a comparison between length effects in the native and foreign languages of bilinguals and show that while these are qualitatively similar, properties of the native language appear to determine processing in the L2.

## Funding

This work was funded through a graduate fellowship to YO by the GRK 1589/2, a graduate research training school of the Deutsche Forschungsgemeinschaft.

### Conflict of interest statement

The authors declare that the research was conducted in the absence of any commercial or financial relationships that could be construed as a potential conflict of interest. The reviewer, Ludovic Ferrand and handling Editor, Jonathan Grainger, declared their shared affiliation, and the handling Editor states that the process nevertheless met the standards of a fair and objective review.

## Footnotes

^1^Across-trial variability in drift rate can be interpreted as due to differences between stimuli. For instance, random across-trial variability in drift rate is sometimes interpreted as reflecting word frequency, or familiarity differences between individuals. As in our model random across-trial variability in drift rate and non-decision time was set to 0 due to computational constraints, our model estimates each participant's average drift rate for each experimental condition only. In other words, here, differences in drift rates and no-decision times reflect the effects of word length and language, but do not account for random variability across items within each subject. Thus we only have random variability in drift rate at the population level, which would account for individual differences in lexical access that are not specific to certain items.

^2^The authors use the leaky accumulator model in this paper, which is equivalent to the drift diffusion model under certain constraints (Bogacz et al., 2006).
