# Supplementary material for: Slower Perception Followed by Faster Lexical Decision in Longer Words: A Diffusion Model Analysis
Source: Front Psychol. 2016 Jan 5;6:1958. doi: 10.3389/fpsyg.2015.01958 (PMC4700557; doi:10.3389/fpsyg.2015.01958)
Supplement: Supplementary file 1 [file Table1.PDF]

frontiers

Supplementary Material

Slower perception followed by faster lexical decision in longer words: a diffusion model analysis

Yulia Oganian[1, 2,3,4*], Eva Froehlich[1, 3,4], Ulrike Schlickeiser[1,3,4], Markus J. Hofmann[3,4,5], Hauke R. Heekeren[1, 3, 4] & Arthur M. Jacobs [1,3,4]

[1] Department of Education and Psychology, Freie Universitaet Berlin, Berlin, Germany,

[2] Bernstein Center for Computational Neuroscience Berlin, Charité, Berlin, Germany,

[3] Dahlem Institute for Neuroimaging of Emotion,Freie Universitaet Berlin, Berlin, Germany,

[4] Center for Cognitive Neuroscience, Freie Universitaet Berlin, Berlin, Germany,

[5] Department of Psychology, Bergische Universitaet Wuppertal, Germany.

*** Correspondence:** [1]Laboratory for biological Psychology and cognitive Neuroscience, Department of Education and Psychology, Freie Universitaet, Habelschwerter Alle 45, 14195 Berlin, Germany. yulia.oganian@ucsf.edu

Supplementary Table S1. Definition of hierarchical diffusion model for JAGS.

```
model {

beta <- 0.5
# alpha
sigma_alpha ~ dunif(exp(-10),2)#
prec_alpha  <- pow(sigma_alpha,-2)

# driftrate
sigma_v0 ~ dunif(exp(-10),2)
prec_v0  <- pow(sigma_v0, -2)
sigma_v1 ~ dunif(exp(-10),2)
prec_v1  <- pow(sigma_v1, -2)

# non-decison time
sigma_t0 ~ dunif(exp(-10),2)
prec_t0  <- pow(sigma_t0, -2)
sigma_t1 ~ dunif(exp(-10),2)
prec_t1  <- pow(sigma_t1, -2)

# variables that change across language blocks
for (clang in 1:2) {
    for (cstim in 1:2)  {
      mu_v0[clang, cstim] ~ dunif(-4,4)
      mu_v1[clang, cstim] ~ dunif(-4,4)
      mu_t0 [clang, cstim] ~ dunif(0.1,2)
      mu_t1 [clang, cstim] ~ dunif(-1,1)
    }
    mu_alpha[clang] ~ dunif(0.5,2)
  }
# variables that change across participants
for (csubj in 1:Nsubj) {
    for (clang in 1:2) {
      alpha[csubj,clang] ~ dnorm(mu_alpha[clang], prec_alpha)
            for (cstim in 1:2) {
                  # driftrate
                  v0[csubj,clang, cstim]~dnorm(mu_v0[clang, cstim], prec_v0)
                  v1[csubj,clang, cstim]~dnorm(mu_v1[clang, cstim], prec_v1)

                  # non-decision time
                  t0[csubj,clang, cstim]~dnorm(mu_t0[clang, cstim], prec_t0)
                  t1[csubj,clang, cstim]~dnorm(mu_t1[clang, cstim], prec_t1)
      }}}

  # dwiener model with variables that change across trials
  for (j in 1:N)
  {t[j] <- t0[subject[j],language[j], stimulus[j]] + t1[subject[j],language[j], stimulus[j]] * length[j]
    nu[j] <- v0[subject[j],language[j], stimulus[j]] + v1[subject[j],language[j], stimulus[j]] * length[j]
    x[j] ~ dwiener(alpha[subject[j], language[j]], t[j], beta, nu[j])
    }}
```

Supplementary Figure S1: Gelman-Rubin statistic for all parameters sampled in the MCMC procedure. R-hat was below 1.1 for all variables, which ensures successful convergence of the MCMC chains to a stationary posterior distribution of model parameters.

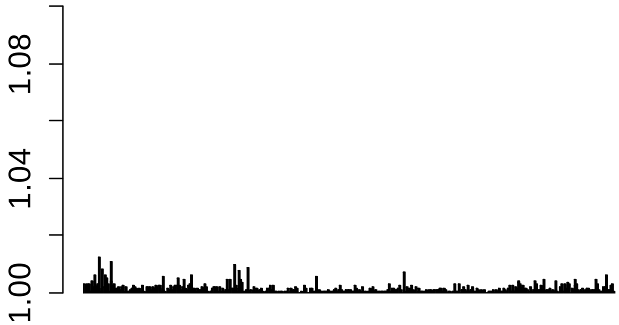

Supplementary Table S2: List of all employed stimuli.

German words

Aal	Butter	Flucht	Holz	normal	Ring	Sofa	Tee
Abt	Couch	Form	Hotel	Nummer	Rock	Sog	Teer
alt	Docht	Format	Jod	Ort	Roman	Spatz	Teil
Angel	Dock	froh	Knirps	Park	rot	Sport	Teller
April	Draht	Frosch	Kreis	Pause	süß	Stand	teuer
Arm	echt	Frost	Krug	Pech	sanft	Start	Text
Art	Effekt	Fuß	kurz	Person	Scherz	Stein	Ticket
Arzt	Eid	Furcht	Lachs	Pfeil	schief	Stern	Tier
Ast	eins	Garten	leicht	Pferd	Schild	stolz	Traum
Bar	eng	Gas	List	Phase	Schnee	stramm	weich
Bau	Erz	Gift	Lust	Plan	schräg	Strand	Wicht
Box	Fee	Golf	Mensch	Poster	Schrei	System	Wild
brav	feucht	Hai	Mut	Rat	See	Taste	Winter
Brief	Finger	Hammer	nass	Rauch	Sieg	Tau	Wut
Bus	Fluch	Hirn	Nest	Rausch	Ski	taub	zart
Zoo	Zug	Zweck	Zyklus				

German pseudo-words

Abl	Colch	Gensch	Kause	Mur	Reman	Siern	teucht
Aht	Domk	Gilf	Kinger	Nas	Reuch	Soff	Texe
Alzt	Druht	Gizt	Kist	Neer	Rier	Spath	Thase
Angeu	eche	Hamber	Knug	Nurrer	Rinf	Spont	Tickit
Anm	Effept	hanft	Kreit	Perfon	Rit	Stalt	Tiste
Apnil	Eld	Hap	kroh	Pferg	Ronk	stalz	Traam
Att	elt	Hest	Krosch	Pfiel	Schenz	Stanf	Tuá
Barten	enns	Hetel	Krost	Pluch	schiel	Stenn	Wiche
Ber	Ert	Hocht	l[illegible]á	Plun	Schilk	strumm	Wister

beuer	Erx	Holp	Lechs	Pnirps	Schlee	Strund	Witd
Birn	Fau	ing	Lormat	Puch	schr[illegible]g	Sug	Wur
Bop	feicht	Irt	Lurcht	Puster	Schreh	Svi	zalt
Bos	Fes	Jol	Lurt	Raesch	seich	Systym	Zau
braw	Flacht	jot	Meller	Rark	Sel	Teif	zaub
Briek	Furm	karz	Mormal	rass	Siel	Tel	Zeg
Butger	Zmeck	Zof	Zyktus				

English words

cage	eel	hammer	normal	purse	start	ticket
car	eerie	hen	numb	rabbit	stein	tier
castle	effect	honor	number	rat	stern	tower
chain	enough	hotel	odd	ring	strand	trace
clasp	fee	hungry	owner	rock	supper	trap
couch	finger	icy	park	Roman	sweat	trash
cure	flower	ill	pause	rot	system	turkey
degree	form	key	pen	sea	tan	tux
dent	format	liar	person	silent	taste	urgent
dew	frost	list	pet	ski	tau	value
dinner	fun	lonely	phase	snake	tea	wallet
dock	garden	lust	plain	sofa	teller	wave
doubt	gas	mind	plan	spit	text	wild
ease	gift	nasty	poster	sport	thin	winter
edge	golf	nest	pot	stand	threat	woman
writer	zero	zoo				

English pseudo-words

adril	cun	enaugh	frast	lat	plar	sterd	trasm
angal	cur	exfect	furse	lig	pon	stirt	troce
ansmer	dar	fel	gest	linner	pooter	strond	turkex
arr	deef	fey	golk	lutt	ret	swaet	tuz
arv	degrae	fiar	gos	mand	ribbet	swort	twap
aystem	dert	fift	haymer	masty	rohan	tam	ursent
boal	deubt	finges	hed	mouch	rong	tastle	vee
boz	dez	flain	hogel	nommer	sapper	tecket	ven
briew	dist	fluwer	hongry	nurmal	sefa	telled	wallit
butmer	dosk	fock	iche	nymb	sident	threet	wape
cee	dus	fonely	ico	ogner	spake	thun	wied
chaan	edde	fonor	jark	oyd	spet	tien	winder
cire	eelie	foom	jee	pauge	spi	tomer	womin
clesp	eese	formit	lan	persan	sprand	toste	wrater
coge	ell	fot	larden	phale	stean	toxt	zalue
zat	zery	zoy					
